# Basic insights into Zika virus infection of neuroglial and brain endothelial cells

**DOI:** 10.1099/jgv.0.001416

**Published:** 2020-04-30

**Authors:** Margit Mutso, James A. St John, Zheng Lung Ling, Felicity J. Burt, Yee Suan Poo, Xiang Liu, Eva Žusinaite, Georges E. Grau, Linda Hueston, Andres Merits, Nicholas J.C. King, Jenny A.K. Ekberg, Suresh Mahalingam

**Affiliations:** ^1^​ Institute for Glycomics, Griffith University, Gold Coast Campus, Southport 4222, Queensland, Australia; ^2^​ Griffith Institute for Drug Discovery, Griffith University, Nathan 4111, Queensland, Australia; ^3^​ Menzies Health Institute Queensland, Griffith University, Gold Coast Campus, Southport 4222, Queensland, Australia; ^4^​ Discipline of Pathology, Bosch Institute, Marie Bashir Institute for Infectious diseases and Biosecurity, School of Medical Sciences, Sydney Medical School, University of Sydney, NSW 2006, Australia; ^5^​ National Health Laboratory Services, University of the Free State, Bloemfontein, South Africa; ^6^​ Institute of Technology, University of Tartu, Tartu, Estonia; ^7^​ Vascular Immunology Unit, Discipline of Pathology, Sydney Medical School, University of Sydney, New South Wales 2050, Australia; ^8^​ Arbovirus Emerging Disease Unit, CIDMLS-ICPMR, Westmead Hospital, Westmead, NSW 2145, Australia

**Keywords:** Zika virus, neural cells, endothelial cells, infectivity, replication kinetics

## Abstract

Zika virus (ZIKV) has recently emerged as an important human pathogen due to the strong evidence that it causes disease of the central nervous system, particularly microcephaly and Guillain–Barré syndrome. The pathogenesis of disease, including mechanisms of neuroinvasion, may include both invasion via the blood–brain barrier and via peripheral (including cranial) nerves. Cellular responses to infection are also poorly understood. This study characterizes the *in vitro* infection of laboratory-adapted ZIKV African MR766 and two Asian strains of (1) brain endothelial cells (hCMEC/D3 cell line) and (2) olfactory ensheathing cells (OECs) (the neuroglia populating cranial nerve I and the olfactory bulb; both human and mouse OEC lines) in comparison to kidney epithelial cells (Vero cells, in which ZIKV infection is well characterized). Readouts included infection kinetics, intracellular virus localization, viral persistence and cytokine responses. Although not as high as in Vero cells, viral titres exceeded 10^4^ plaque-forming units (p.f.u.) ml^−1^ in the endothelial/neuroglial cell types, except hOECs. Despite these substantial titres, a relatively small proportion of neuroglial cells were primarily infected. Immunolabelling of infected cells revealed localization of the ZIKV envelope and NS3 proteins in the cytoplasm; NS3 staining overlapped with that of dsRNA replication intermediate and the endoplasmic reticulum (ER). Infected OECs and endothelial cells produced high levels of pro-inflammatory chemokines. Nevertheless, ZIKV was also able to establish persistent infection in hOEC and hCMEC/D3 cells. Taken together, these results provide basic insights into ZIKV infection of endothelial and neuroglial cells and will form the basis for further study of ZIKV disease mechanisms.

## Introduction

Zika virus (ZIKV) is a recently emerged mosquito-borne virus belonging to the genus *Flavivirus*, family *Flaviviridae*. The virus was initially isolated from a rhesus monkey in the Zika forest near Entebbe, Uganda, during yellow fever investigations in 1947 and was subsequently isolated from wild-caught *Aedes* spp. mosquitoes collected in Uganda in 1948 [1]. The recent emergence of this virus as a cause of larger outbreaks of disease was first reported in 2007 when an outbreak of ZIKV was identified on Yap Island, Federated States of Micronesia, in the southwestern Pacific Ocean [2]. Three-quarters of the population of Yap Island were estimated to be infected during the outbreak, with the majority of the patients presenting with mild disease [3]. In October 2013 the virus was identified as the cause of an outbreak of dengue-like illness in French Polynesia, located in the South Pacific [4, 5]. Thousands of suspected cases of ZIKV infection were reported during the outbreak, with most patients presenting with mild disease, fever, arthralgia, maculopapular rash and conjunctivitis. During these outbreaks, an increase of neurological complications in the form of Guillain–Barré syndrome (GBS) in ZIKV-infected patients and microcephaly associated with ZIKV infection during pregnancy were noted [6, 7].

The pathogenesis of disease caused by ZIKV, including the mechanisms of neuroinvasion and host cell responses to infection, are currently not clearly delineated. The pathway by which ZIKV gains access to the central nervous system (CNS) is unknown. The mechanism of neuroinvasion may involve multiple routes, as is seen with other neurotropic flaviviruses, such as West Nile virus, for which hypotheses of both haematogenous and transneural entry have been proposed [8, 9].

Olfactory ensheathing cells (OECs), the glial cells of the primary olfactory nervous system, are found in the olfactory nerve and bulb, and have crucial roles in the regeneration of olfactory axons, which occurs throughout life. Transneuronal transmission of neurotropic virus such as rabies virus [10] has been shown to involve the olfactory system, but it remains unknown whether other neurotropic viruses such as ZIKV can enter the CNS via this path. Further, whilst it is known that OECs are highly phagocytic cells that can phagocytose microorganisms and be pathogen hosts [11–14], their roles in virus dissemination or as immunoregulatory cells *in vivo* are not clearly defined.

Another potential neuro-invasion model for micro-organisms that has been proposed is crossing the blood–brain barrier (BBB). The BBB prevents virus circulating in blood from entering the brain. The human cerebral microvascular endothelial cell line (hCMEC/D3) is a stable, easily grown BBB cellular model used in a wide range of research areas, including passage of infectious micro-organisms across the BBB [15–19]. There are reports showing that hCMEC/D3 cells are susceptible to ZIKV infection, leading to the speculation that ZIKV has the ability to cross the BBB [18, 20].

In the present study, the permissiveness of human and mouse neuroglial cells, including OECs and hCMEC/D3s, to ZIKV strains belonging to Asian genotypes and the highly adapted MR766 was investigated. We show that brain endothelial cells and neuroglial cells are permissive for ZIKV infection, may potentially serve as a persistent reservoir of infection, and could contribute to CNS inflammation through the production of pro-inflammatory chemokines.

## Methods

### Cells

Immortalized mouse olfactory ensheathing cells (mOECs) [21] and human olfactory ensheathing cells (hOECs) [22] were maintained in Dulbecco’s modified Eagle’s medium (DMEM) with 10 % foetal calf serum (FCS). Vero cells (African green monkey epithelial cells; ATCC, CCL-81) were maintained in DMEM with 10 % FCS. Human brain endothelial cells (hCMEC/D3) [23] were maintained in endothelial cell growth basal medium (EBM-2 medium) with 5 % FCS, 1.4 µm hydrocortisone, 5 μg ml^−1^ ascorbic acid, 1 % chemically defined lipid concentrate, 10 mM HEPES and 1 ng ml^−1^ basic fibroblast growth factor. All cells were cultured at 37 °C in a humidified atmosphere of 5 % CO_2_ and were passaged at 2-day intervals after attaining 70–75% confluency.

### Virus

Three ZIKV strains were used. (i) MR766 (Uganda), an African genotype, is a rhesus monkey origin virus isolate from the Centers for Disease Control and Prevention (CDC), which is strongly adapted to mice. While MR766 does not represent the natural African lineage due to it being highly adapted, it nevertheless has been used in many other studies, thus allowing a good comparison with those studies. (ii) BeH89015, an Asian genotype, derived from an infectious clone based on the sequence of the Brazilian strain [24]. (iii) PRVABC59, an Asian genotype, is a human isolate from a Puerto Rico patient [25] and was kindly provided by Dr Jill Carr, Flinders University, Australia. These viruses were propagated and plaque-titred in Vero cells.

### Plaque assay

Viral titres were determined by plaque assay on Vero cells. Briefly, Vero cells were seeded in 12-well plates. Tenfold dilutions of each virus stock were prepared in culture medium and 100 µl of each dilution was added to the cells. Cells were incubated with virus dilution for 1 h at 37 °C and then the inoculum was removed and the cells were overlaid with 1 ml of DMEM supplemented with 2 % FCS containing 0.8 % carboxymethyl cellulose (Sigma Life Science). Cells were incubated at 37 °C for 6 days prior to staining with crystal violet fixing solution (0.1 % crystal violet in 20 % ethanol and 3.7 % formaldehyde). Plaques were counted and the virus titre expressed as plaque-forming units (p.f.u.) ml^−1^. All multiplicity of infection (m.o.i.) values used in subsequent experiments were calculated based on virus titres in Vero cells.

### Growth curve analysis

Multistep growth curves were performed using mOEC, hOEC, hCMEC/D3 and Vero cell lines. Briefly, 10^5^ cells were seeded in 12-well plates and infected with ZIKV at an m.o.i. of 0.1 in serum-free media. Cells were incubated for 1 h, the inoculum was removed and the cells were washed twice with phosphate-buffered saline (PBS). The cells were overlaid with 0.5 ml of complete media and incubated at 37 °C. Cell culture media were collected at 0, 0.5, 1, 2, 3, 4, 5 and 6 days post-infection (p.i.). At each time point, all media were removed and replaced with fresh medium. Virus titres were determined by plaque assay.

### Immunofluorescence microscopy

Cells were cultured on glass coverslips, infected at an m.o.i. of 1 in serum-free medium for 1 h, washed once with PBS and covered with complete growth medium. Mock-infected cells were used as controls. At indicated time points, the cells were washed with PBS, fixed with 4 % paraformaldehyde and permeabilized with 0.1 % Triton X-100 for 2 min at room temperature. Fixed cells were washed with PBS and blocked with 5 % FCS in PBS. Cells were immunolabelled using the following primary antibodies: rabbit anti-NS3 (obtained in-house) at 1 : 5000 dilution [24], mouse anti-flavivirus group antigen antibody, clone D1-4G2-4-15 (Millipore, USA), monoclonal anti-dsRNA antibody (English and Scientific Consulting; Szirak, Hungary) or Golgi marker mouse anti-58k (Abcam, USA) at 1 : 200 dilution for 1 h or PDI-FITC (Thermo Fisher, USA). Following the removal of primary antibodies, cells were washed and treated with the respective anti-rabbit/mouse Alexa-568/488/647-conjugated goat antibodies (Life Technologies); 4′,6′-diamidino-2-phenylindole (DAPI) (Life Technologies) was used to counterstain nuclei. Slides were mounted using SlowFade Gold reagent. Immunofluorescence images were obtained and analysed using a Nikon A1R+ confocal microscope. Images were analysed using Imaris software.

### Viral infectivity

To determine the infectivity of ZIKV in the selected cell lines, confluent monolayers of each cell type were infected at an m.o.i. of 1. Samples were harvested at 16 h post-infection (p.i.) and stained with LIVE/DEAD cell viability dye (Life Technologies). Cells were fixed in 1 % paraformaldehyde in PBS at room temperature for 15 min, washed twice with FACS buffer (PBS with 2 % FBS) and permeabilized using 0.2 % Tween 20 in FACS buffer for 15 min at 37 °C. Cells were labelled with rabbit anti-NS3 primary antibody, diluted 1 : 5000 in 0.1 % Tween 20 in FACS buffer (diluent) for 30 min on ice and washed twice in diluent. The primary antibody was detected using anti-rabbit Alexa 568 antibody in 0.1 % Tween 20 in FACS buffer. Samples were examined using the BD LSR Fortessa Cell Analyser and the resulting data were analysed with FlowJo software. The live and NS3-positive population of each cell line was determined and used as an indication of the efficiency of viral infection.

### Cell viability assay

The viability of infected cells was determined using a cell proliferation assay (MTT, Sigma-Aldrich, USA). Briefly, 96-well plates were seeded with 5×10^4^ cells per well. The cells were infected with MR766 or BeH819015 strain at an m.o.i. of 1. After 1 h the media was replaced with 200 µl of complete medium. At 1, 3, 5 or 10 days p.i. 20 µl of MTT reagent (5 mg ml^−1^ in PBS) was added into each well; cells were incubated for 2 to 4 h until a purple precipitate become visible. The medium was removed, the precipitates were dissolved by adding 100 µl of DMSO and absorbance was measured at 560 nm.

### Generation of persistently infected cells, detection of virion production and effect of ZIKV on cell proliferation

Mouse and human OECs and hCMEC/D3 cells were infected as described above. Infected cultures were maintained for 2 months, with 1 : 5 dilution of the culture when reaching 70–80 % confluency. The cell lines obtained using this procedure are referred to as mOEC_MR766, hOEC_MR766, hCMEC/D3_MR766, mOEC_BeH819015, hOEC_BeH819015, hCMEC/D3_BeH819015, mOEC_PRVABC59, hOEC_PRVABC59 and hCMEC/D3_PRVABC59. To analyse the presence of ZIKV RNA in mOEC_MR766, hOEC_MR766, hCMEC/D3_MR766, mOEC_BeH819015, hOEC_BeH819015, hCMEC/D3_BeH819015, mOEC_PRVABC59, hOEC_PRVABC59 and hCMEC/D3_PRVABC59 cells, the cells were seeded in six-well plates at a density of approximately 10^5^ cells/well. After 24 h incubation, cells were washed twice with PBS and replaced with 2 ml of fresh media. After another 24 h incubation cells were collected and total RNA was extracted for determination of virus genome copy number. To analyse the growth of ZIKV-infected cells, hOEC, hOEC_MR766, hOEC_PRVABC59, hCMEC/D3, hCMEC/D3_MR766 and hCMEC/D3_PRVABC59 cells were seeded in six-well plates at a density of 50 000 cells/well. Day 1, 3 and 5 post-seeding total cell numbers in the well were counted using the standard haemocytometer cell counting method according to protocol. Briefly, cells were trypsinized and collected into 1 ml medium to obtain a cell suspension. Fifty microlitres of cell suspension was mixed with 50 µl of 0.4 % trypan blue solution and was applied into the haemocytometer chamber. The total cell number was counted using the following formula: cells/ml=(total cells counted/number of boxes counted)×dilution factor×10 000×total sample volume.

### RNA extraction and quantitative reverse-transcription PCR (RT-qPCR)

Total RNA were extracted from persistently infected cells using the RNeasy kit (Qiagen, Germany) according to the manufacturer’s protocol. The obtained RNA was reverse-transcribed using random nanomer primers and Moloney murine leukaemia virus reverse transcriptase (Sigma-Aldrich). Serial dilutions of pCCI-SP6 ZIKV plasmid [24] were used to generate a gene copy standard curve. Viral genome copy number was determined by RT-qPCR using SsoAdvanced universal probes supermix (Bio-Rad) in a 12.5 µl reaction and primers targeting the NS5 region (MR766, 5′AAATACACATACCAAAACAAAGTGGT and 5′TCCACTCCCTCTCTGGTGTTG) or envelope region (PRVABC59 and BeH918015, 5′ AGATGTCGGCCCTGGAGTTC and 5′ TTGCCACACCGTCCTTGAGG). All reactions were performed in 96-well plates using a Bio-Rad CFX96 Touch real-time PCR detection system. The samples were amplified using the following conditions: 95 °C for 15 min, and 40 cycles of 94 °C for 15 s, 55 °C for 30 s and 72 °C for 30 s. A dissociation curve was acquired using CFX Manager software and used to determine the specificity of the amplified products. The copy numbers of the amplified products were interpolated from the standard curve using GraphPad Prism software.

### Cytokine assay

Media collected from infected hOEC and hCMEC/D3 cells were assessed for CCL2, CCL3, CCL4, CCL5 and CXCL8 using enzyme-linked immunosorbent assay (ELISA) (R and D Systems) as specified by the manufacturer.

### Statistical analysis

All statistical analyses were performed with GraphPad Prism software. Statistical differences were analysed using two-way analysis of variance (ANOVA) with Bonferroni’s post hoc and one-way ANOVA with a Tukey’s post hoc test.

## Results

### ZIKV strains replicate in brain endothelial and neuroglial cells

**Fig. 1.**
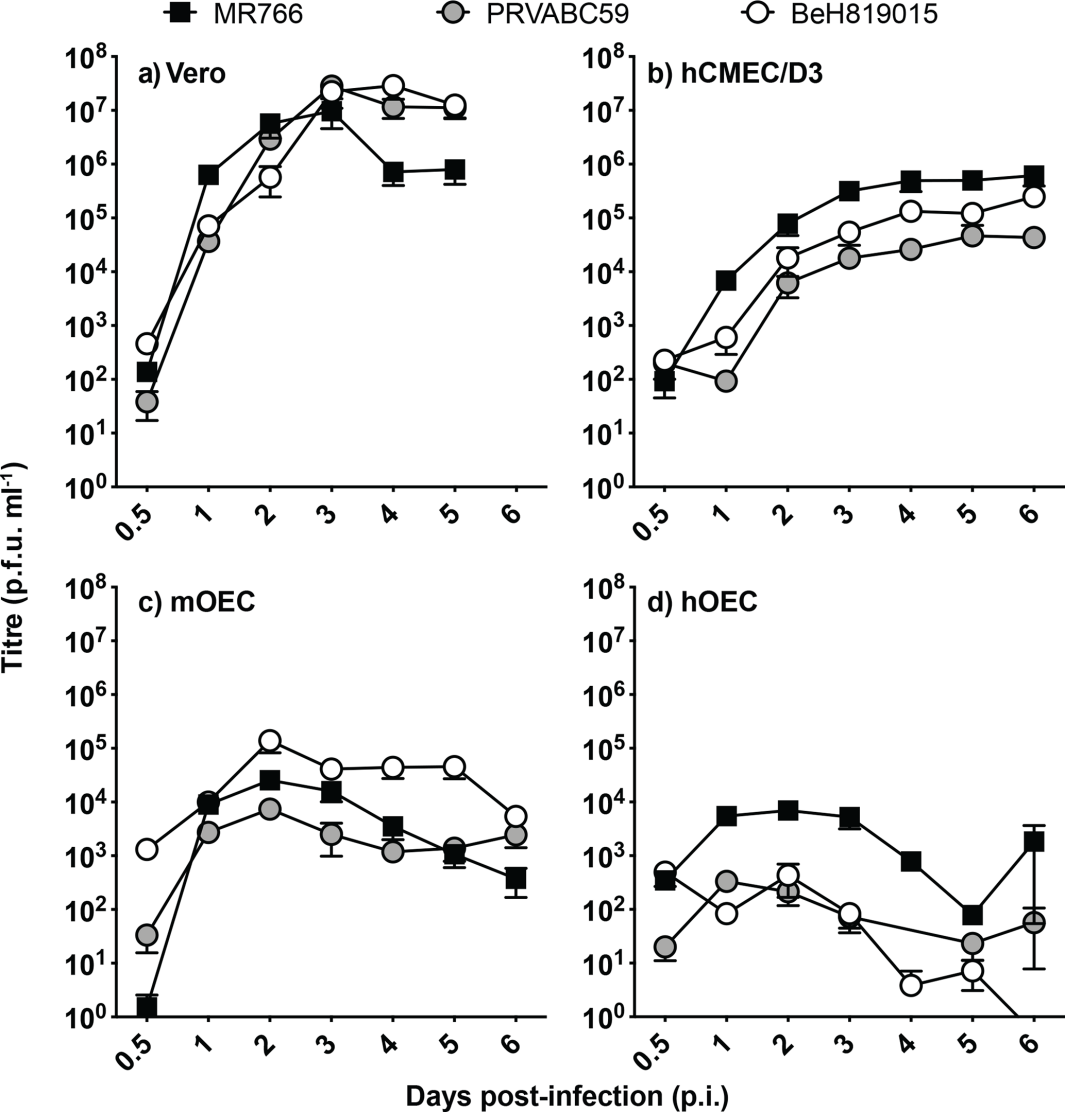
ZIKV replication in non-neural and neuroglial cells. Multistep growth curves of ZIKV (m.o.i. 0.1) in (a) kidney epithelial (Vero) cells, (b) brain endothelial cells (hCMEC/D3), (c) mouse neuroglia cells (mOEC) and (d) human neuroglia (hOEC) cells. Virus titres were determined using plaque assay. For each time point, the mean±sem (*n*=7) is presented. Data were derived from three independent experiments. The dotted line indicates the detection limit of the assay (30 p.f.u. ml^−1^). The 0.5 h time point for PRVABC59 was below the limit of detection.

To characterize infection by different ZIKV strains (MR766, PRVABC59 and BeH819015), brain endothelial cells (hCMEC/D3) and neuroglial olfactory ensheathing cells (hOEC and mOEC) were infected at an m.o.i. of 0.1. Vero cells, for which ZIKV infection is well characterized, were used as a positive control. Although not reaching titres as high as those in Vero cells (Fig. 1a; peaked ~10^7^ p.f.u. ml^−1^ at 3 days p.i.), all ZIKV strains replicated in brain endothelial cells and neuroglial cells, with the highest titres being observed in hCMEC/D3 (Fig. 1b; peaked ~10^6^ p.f.u. ml^−1^ at 6 days p.i.) followed by mOEC (Fig. 1c; peaked around 10^5^ p.f.u. ml^−1^ at 2 days p.i.). In hOEC cells, titres clearly above the detection limit were only observed for the MR776 strain (Fig. 1d; peaked ~10^4^ p.f.u. ml^−1^ at 2 days p.i.). These observations show that both African and Asian ZIKV strains are able to infect and replicate in neuroglial and brain endothelial cells.

**Fig. 2.**
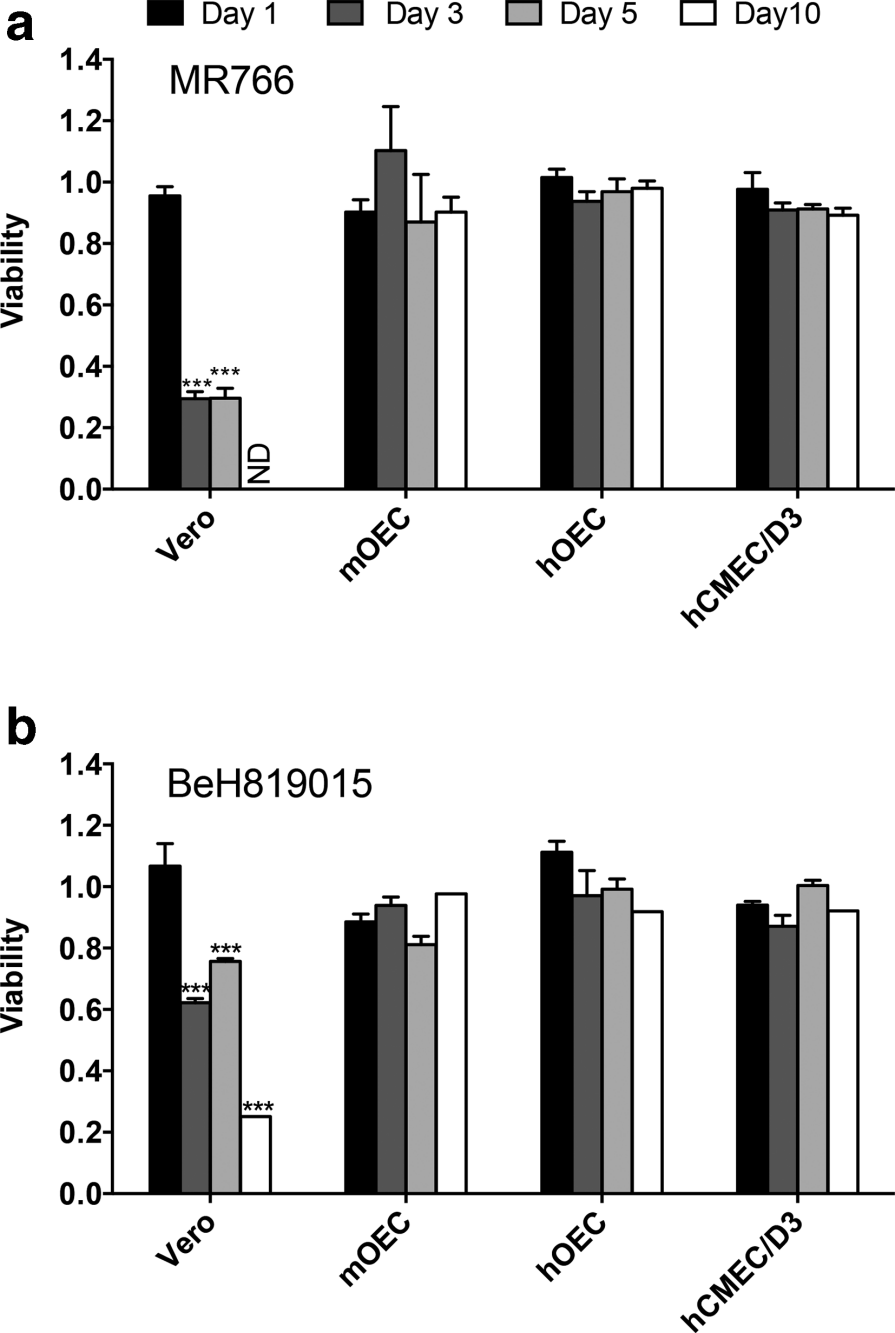
Viability of ZIKV-infected cells. Vero, mOEC, hOEC and hCMEC/D3 cell lines were infected at an m.o.i. of 1 with (a) ZIKV MR766 or (b) BeH819015 or were mock-infected. At 1, 3, 5 and 10 days p.i. cell viability was determined by MTT assay. The obtained values were normalized to mock-infected cells (set as 1). Each bar represents the mean±sem of two independent experiments performed in triplicate. nd, not detectable; ***P<0.001. The viability of infected cells was compared to that of uninfected cells using two-way ANOVA with Bonferroni’s post-test (*n*=6).

With the exception of mOEC cells, MR766 appeared to be the fastest replicating ZIKV strain, with titres that were consistently higher at 2 days p.i. compared to PRVABC59 and BeH819015(Fig. 1a,b d). Consistent with this observation, MR766 also induced more prominent cytopathic effect (CPE) in Vero cells than BeH819015: the viability of MR766-infected cells was reduced to ~25 % by 3 days p.i., while, in contrast, the viability of BeH819015-infected cells remained at >60 % even at 5 days p.i. (Fig. 2a, b). In contrast to Vero cells, both MR766 and BeH819015 strains had no significant effect on the viability of neuroglial or brain endothelial cells (Fig. 2a, b). Combined, these findings suggest that neuroglial and brain endothelial cells may harbour an intrinsic mechanism of suppressing or controlling infection, viral replication and virus-induced cytotoxicity.

### Low efficiency of ZIKV infection in neuroglial and endothelial cells

**Fig. 3.**
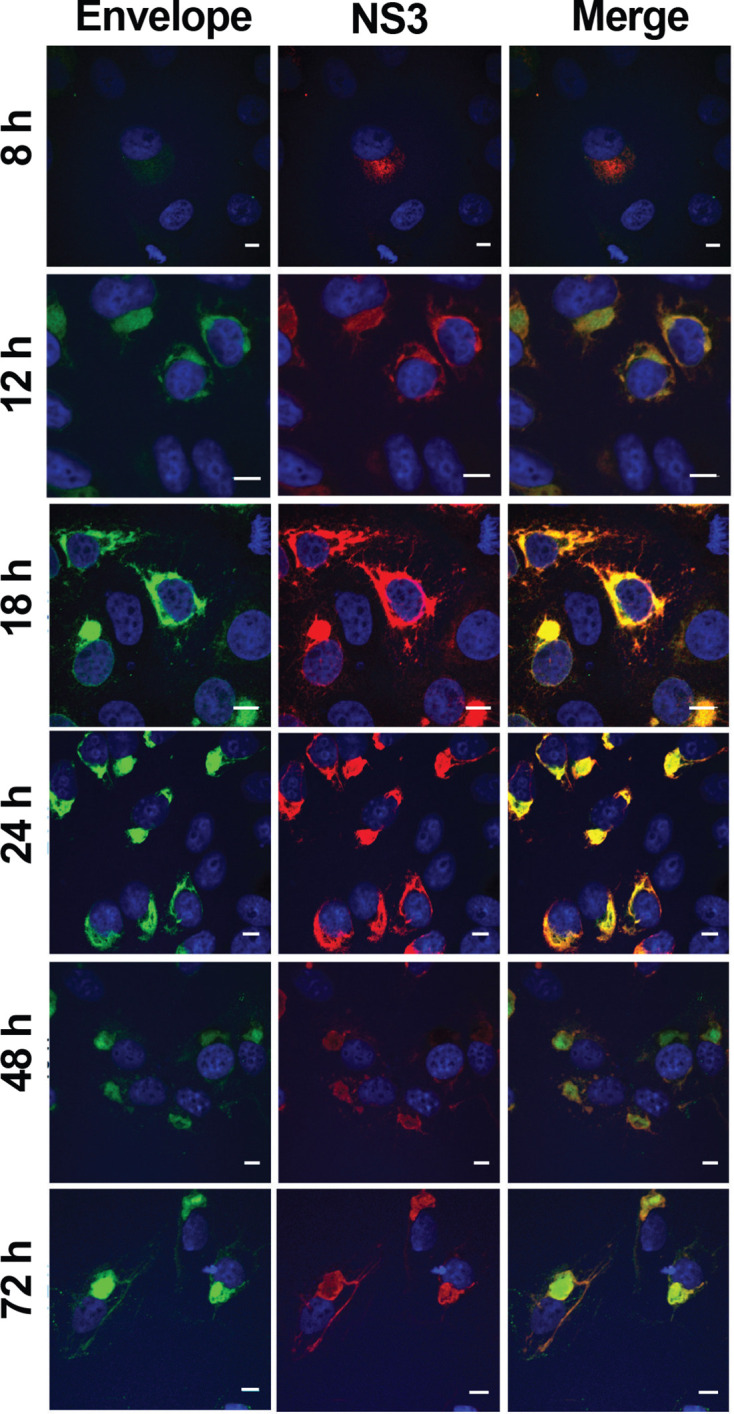
Localization of ZIKV proteins in infected cells. Vero cells were infected with ZIKV MR766 at an m.o.i. of 1. At 8, 12, 16, 24, 48 and 72 h p.i. cells were fixed and analysed using an immunofluorescent assay for the localization of envelope protein (green) and NS3 (red). DAPI (blue) was used to counterstain nuclei. The scale bars represent 10 µm.

**Fig. 4.**
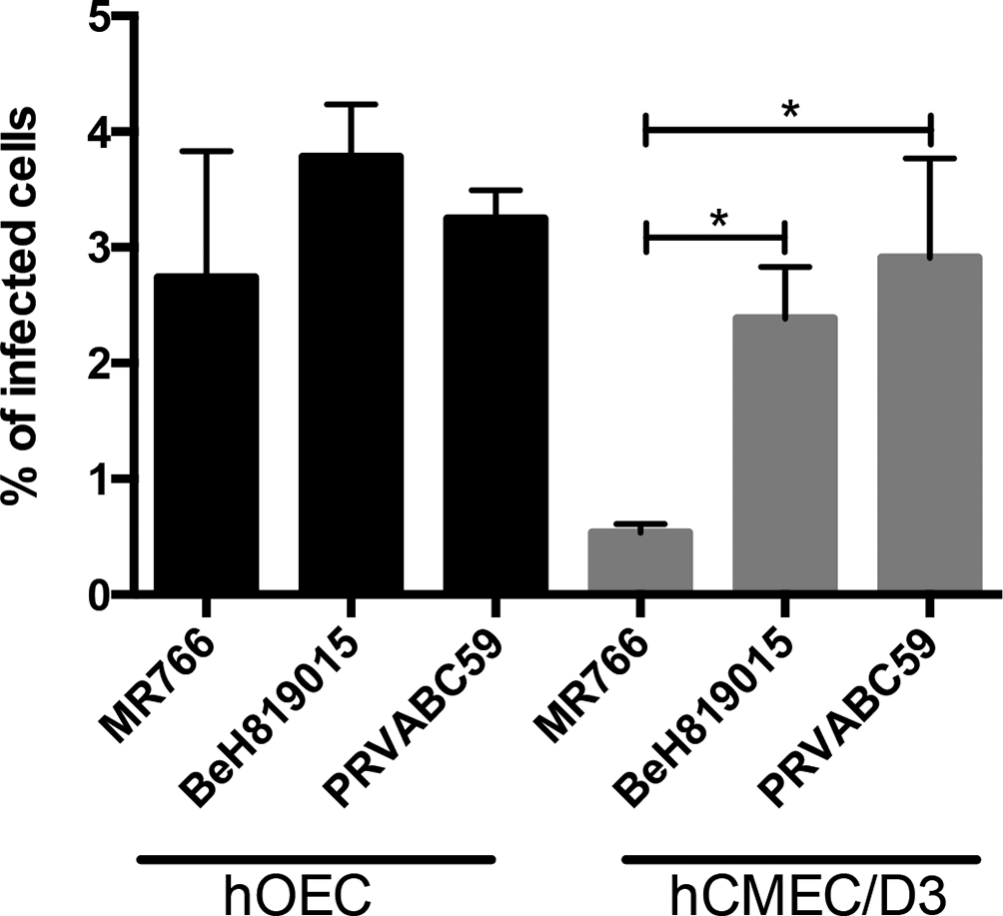
ZIKV infectivity in hOEC and hCMEC/D3 cells. hOEC and hCMEC/D3 cells were infected with ZIKV at an m.o.i. of 1 (calculation based on titre from Vero cells). The cells were harvested at 16 h p.i. and ZIKV NS3 expression was measured by staining with anti-NS3 antibody and FACS analysis. At least 100 000 events were captured per sample. Each bar represents the mean±sem of two independent experiments performed in triplicate. *P<0.01.

We next determined ZIKV infection efficiency in different neuroglial cell lines by detecting the percentage of cells that were positive for ZIKV non-structural protein 3 (NS3). The viral growth curve revealed that in Vero cells the release of new viral particles only occurred after 12 h p.i. (Fig. S1, available in the online version of this article) and the highest levels of NS3 were observed at 18–24 h p.i. (Fig. 3). Therefore, infected hOEC and hCMEC/D3 cells were harvested at 16 h p.i.; at this time point only primarily infected cells had detectable expression of NS3. Flow cytometry revealed that the infection efficiency of all ZIKV strains in the neuroglial cells (hOEC) and endothelial cells (hCMEC/D3) was much lower than could be counted from the m.o.i. calculated based on titres obtained using Vero cells. Unlike in the growth curve experiment (Fig. 1d), all three strains performed similarly in hOEC cells and infected approximately 3 % of the total cell numbers (Fig. 4). Interestingly, in hCMEC/D3 cells both Asian strains showed significantly higher infectivity (~3 % infected cells) compared to the MR766 strain (~0.5 % of infected cells). These results clearly show a much lower susceptibility to virus infection in neuroglial and brain endothelial cells compared to non-neural Vero cells; this finding is consistent with the observed lower growth efficiency (Fig. 1). Furthermore, there is no clear correlation between efficiency of infection and efficiency of replication (virion production) in neuroglial and brain endothelial cells. Thus, MR766 has the lowest infection efficiency in hCMEC/D3 cells (Fig. 4) and yet it produced more virions than any of the Asian strains (Fig. 1b). These data, again, may also suggest that intrinsic mechanism(s) responsible for reduced viral entry or/and replication and/or virion formation/release are present in neuroglial and endothelial cells.

### ZIKV envelope protein, NS3 and dsRNAs are localized in ER membranes

To study the intracellular localization of ZIKV NS3 and envelope proteins, ZIKV-infected Vero cells were examined by immunofluorescence assay (IFA). Viral proteins were visualized at 8, 12, 18, 24, 48 and 72 h p.i. (Fig. 3). At 12 h p.i., the NS3 and envelope proteins were distributed evenly in the cell cytoplasm. The highest levels of envelope and NS3 protein were detected at 18 h p.i. and 24 h p.i. At these time points, localization of both NS3 and envelope protein appeared to be in cytoplasmic membranes that may represent endoplasmic reticulum (ER) and/or Golgi apparatus. NS3, albeit at reduced levels, was also detected at both 48 and 72 h p.i (Fig. 3). At 72 h, there were signs of cell death (Fig. 3), an observation consistent with the results from the viability assay (Fig. 2).

**Fig. 5.**
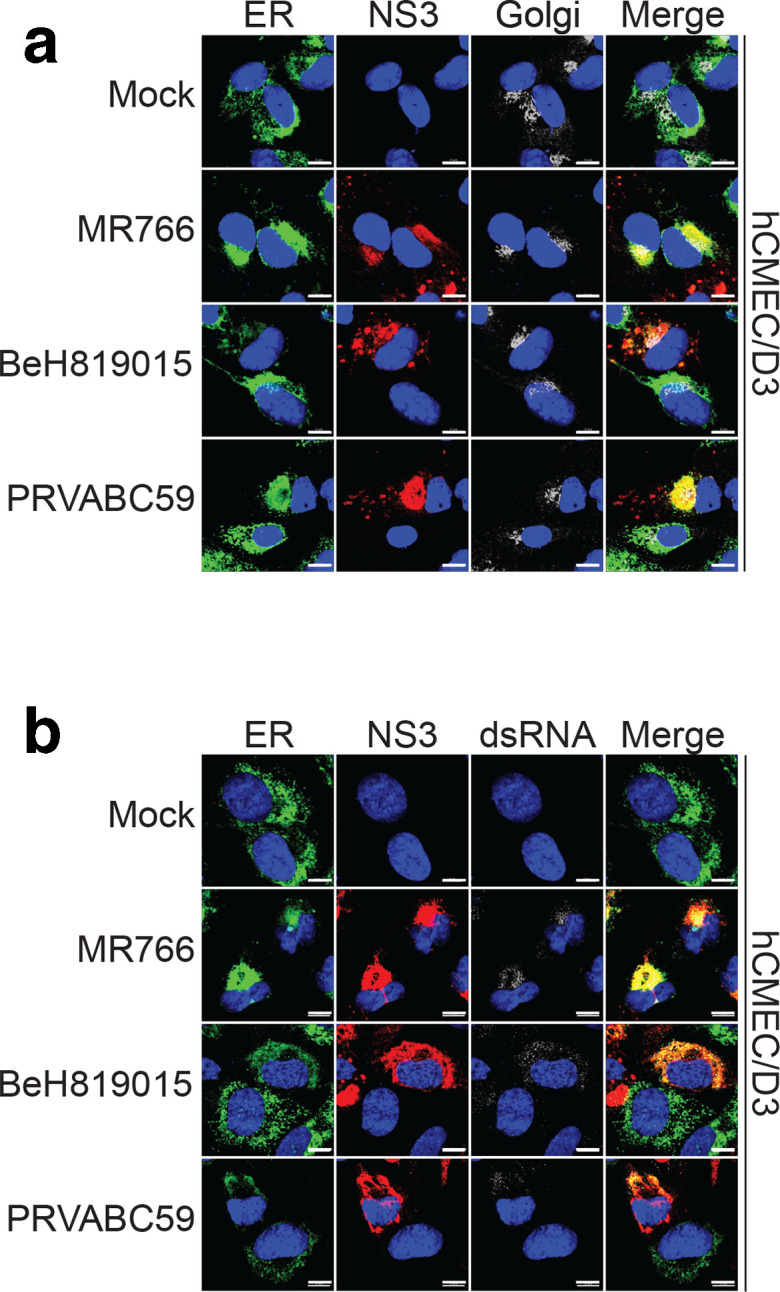
Localization of NS3 and dsRNA in ZIKV-infected hCMEC/D3 cells. hCMEC/D3 cells were infected with different strains of ZIKV at an m.o.i. of 1 for 24 h. (a) Cells were stained with antibodies against ER marker (green), ZIKV NS3 (red) and Golgi marker (silver). Nuclei were counterstained with DAPI (blue). (b) Cells were stained with antibodies against endoplasmic ER marker (green), ZIKV NS3 (red) and dsRNA (silver). Nuclei were counterstained with DAPI (blue). Scale bar is 10 µm.

Next, hCMEC/D3 cells infected with different ZIKV isolates were examined by IFA at 24 h p.i. using antibodies against Golgi apparatus (65K protein), ER membranes (PDI), NS3 and dsRNA that served as a marker for viral RNA replication. Consistent with previous observations NS3 was localized in the cytoplasm (Fig. 5a, b). The widespread punctate staining pattern of dsRNA overlapped in some areas with NS3 staining and showed clear co-localization with ER membrane (Fig. 5b), a site of likely localization of ZIKV replication complexes. Overlap of NS3 was also observed with Golgi marker, but not to the same extent as for ER marker (Fig. 5a). The IFA results obtained for ZIKV infected Vero, hOEC and mOEC cells (Fig. S2) were almost identical to those obtained for hCMEC/D3 cells (Fig. 5).

### hCMEC/D3, hOEC and mOEC cells can be persistently infected by different ZIKV strains

**Fig. 6.**
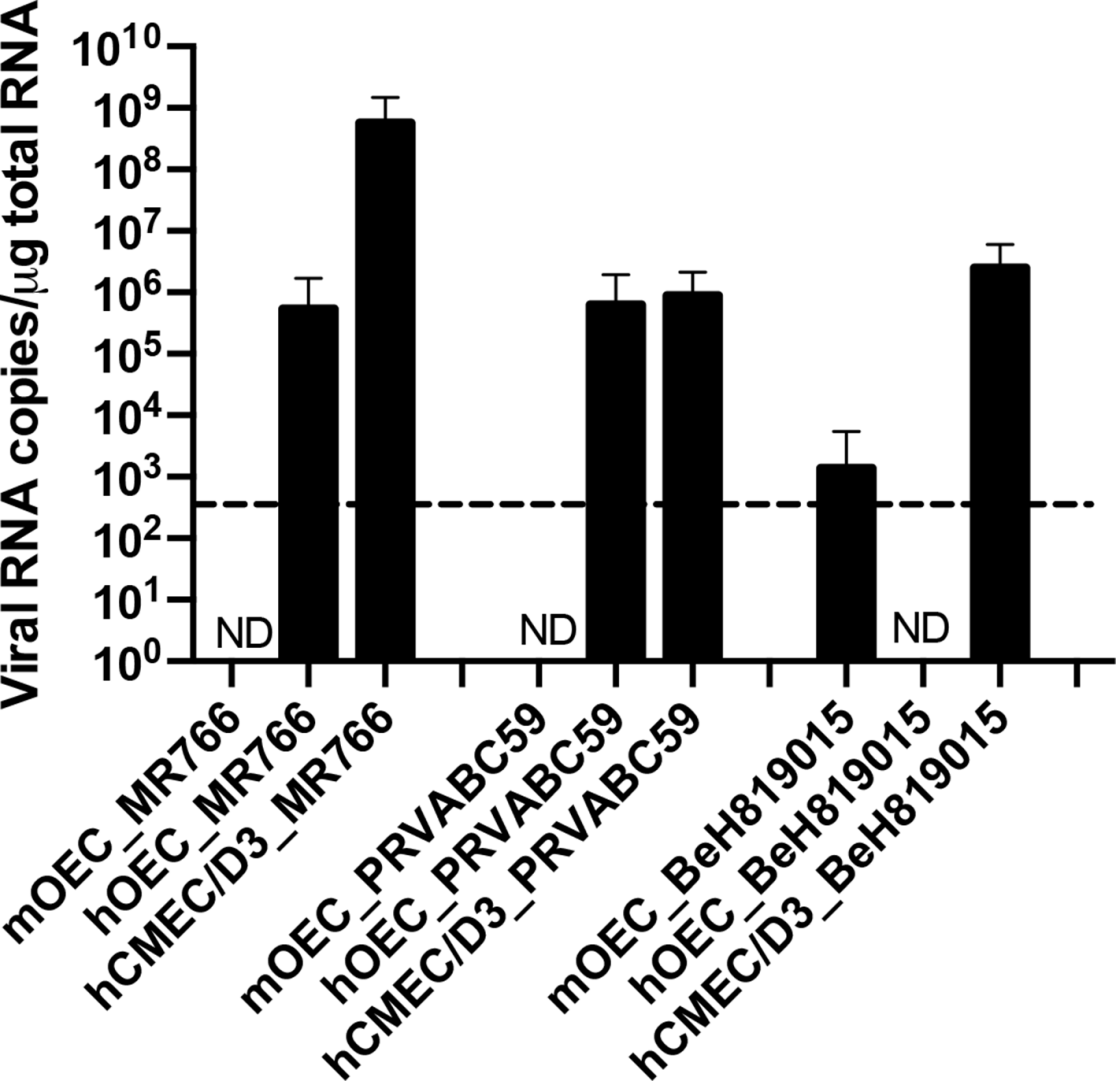
ZIKV can establish persistent infection in mOEC, hOEC and hCMEC/D3 cell cultures. mOEC, hOEC and hCMEC/D3 cell cultures were infected with three different ZIKV strains for 2 months. After this, cells were collected, total RNA was isolated and viral genome copy numbers were determined using RT-qPCR. The data represent the mean±sem from two independent experiments. nd, not detected. The dotted line indicates the detection limit of the assay (600 viral RNA copies µg^−1^ total RNA).

To examine the virus persistence in neuroglia and human brain endothelial cell cultures, the viral RNA copy numbers in hCMEC/D3, hOEC and mOEC cells infected with MR766, PRVABC59 and BeH819015 for 2 months were quantified using RT-qPCR (Fig. 6). The viral genome copy numbers were high in MR766- and PRVABC59-infected hCMEC/D3 and hOEC cells. For BeH819015 the viral copy number was only high in hCMEC/D3 cells. BeH819015 was also detected in mOEC cells, albeit at low levels. Release of infectious virions was only detected for hCMEC/D3 infected with any of the ZIKV strains (data not shown). These data indicated that all three cell lines can become persistently infected. Furthermore, at least hCMEC/D3 cell line produced ZIKV virions after 2 months of infection. Analysis of the percentage of NS3-positive cells using staining with anti-NS3 antibody and FACS analysis revealed that NS3 levels were only above the detection limit in some of the cells; the percentage of NS3-positive cells showed large variation (from 2–58 %) depending on cell line and clone. A correlation between virus titre and the percentage of NS3-positive cells in the culture was also observed. These data indicate that cells do not harbour ZIKV in a uniform manner in persistently infected cultures and contain a variable percentage of cells in which the presence of ZIKV cannot be revealed.

### ZIKV persistent infection leads to reduced cell proliferation in hCMEC/D3 cells

**Fig. 7.**
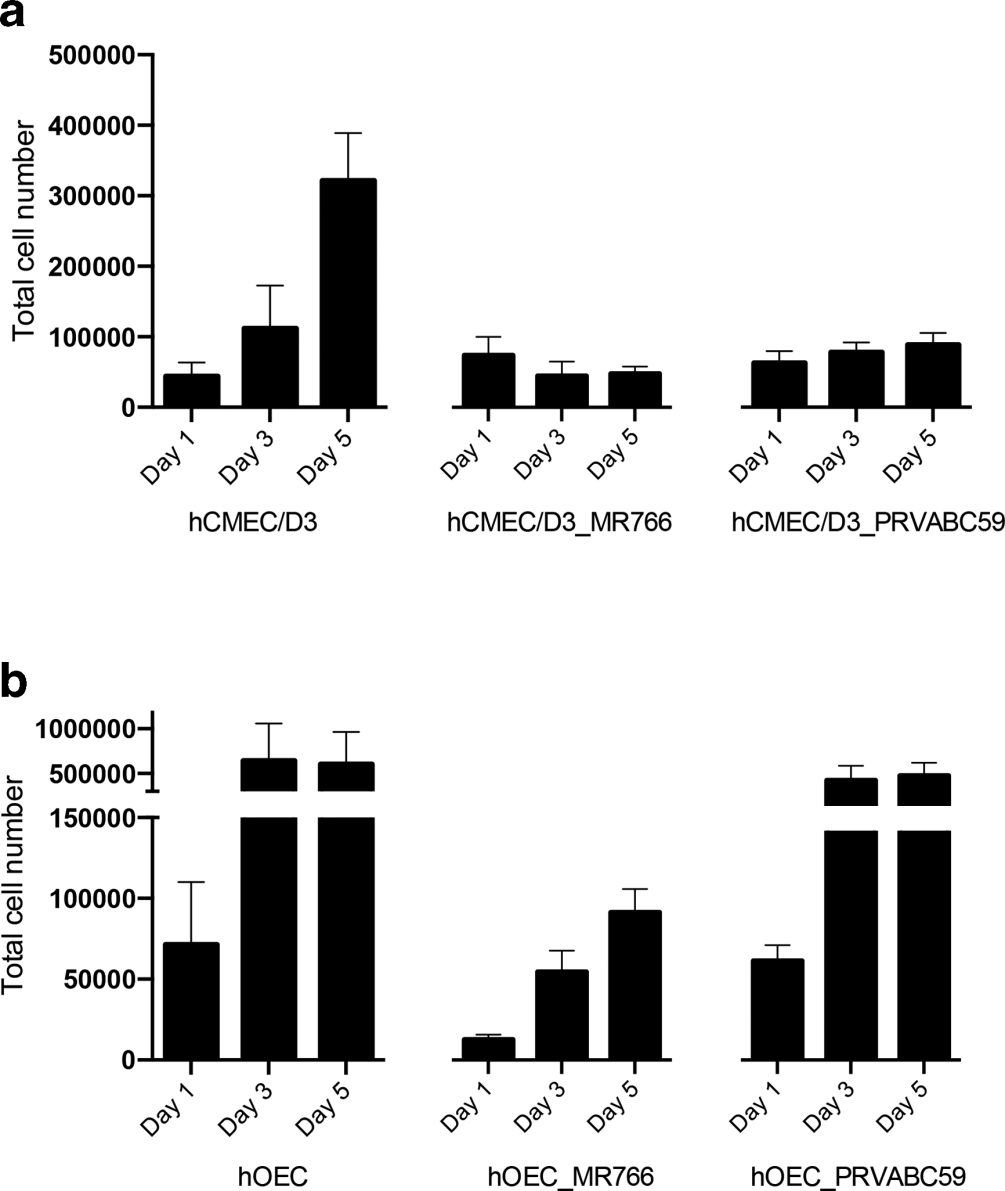
Proliferation of uninfected and 2-month persistently infected cells. (a) hCMEC/D3 (uninfected), hCMEC/D3_MR766 and hCMEC/D3_PRVABC59 cells or (b) hOEC (uninfected), hOEC_MR766 and hOEC_PRVABC59 cells were seeded on 24-well plate at a density of 50 000 cells per well. At days 1, 3 and 5 post-seeding the total cell numbers were counted. The data represent the mean±sem from three independent experiments.

MTT assays did not reveal any cytotoxic effect of ZIKV acute infection in brain endothelial and neuroglial cells (Fig. 2). Interestingly, all these cells still produced infectious virions, in some cases even at 6 days p.i. (Fig. 1b–d), and, at least in the case of hCMEC/D3 cells, continued to do so 2 months later. To understand the effect of ZIKV persistence in these cells, we compared the proliferation of hCMEC/D3 and hOEC cells that had been preinfected for 2 months with ZIKV-MR766 or ZIKV-PRVABC59 with the growth of the same type of uninfected cells over a 5 day period. It was found that the proliferation of hCMEC/D3 cell lines infected with either ZIKV strain was clearly lower than that of uninfected cells, and the differences become prominent at day 3 and further increased by day 5 after the cells had been seeded (Fig. 7a). Similarly, the proliferation of hOEC_MR766 cells was slow. In addition, these cells exhibited poor attachment, with the result being a loss of ~50 % of cells observed by day 1. In contrast, persistent infection of hOEC cells by PRVABC59 had no detectable effect on cell proliferation (Fig. 7b).

### Chemokine levels in ZIKV-infected cells

**Fig. 8.**
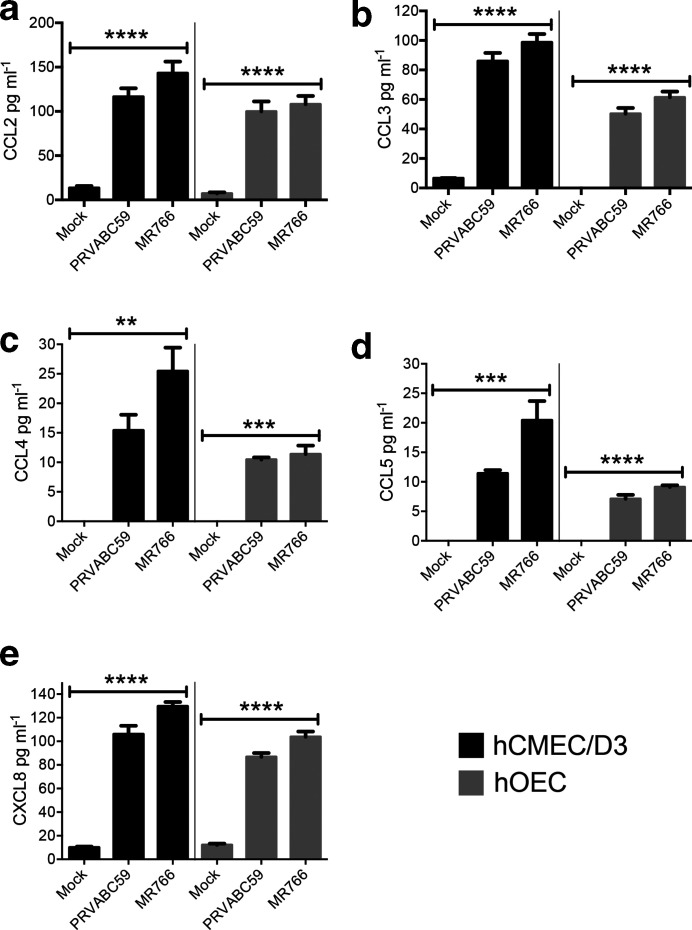
Chemokine levels in ZIKV MR766- or PRVABC59-infected hCMEC/D3 and hOEC cell cultures. hCMEC/D3 and hOEC cells were infected with ZIKV MR766 or PRVABC59 at an m.o.i. of 1; control cells were mock infected. At 24 h p.i. growth media were collected, clarified by centrifugation and assessed for levels of CCL2, CCL3, CCL4, CCL5 and CXCL8. The data represent the mean±sem from two independent experiments. **P<0.01, ***P<0.001 and ****P<0.0001 [infected cells (*n*=4) compared to uninfected cells (*n*=2)]. *P* values were determined by one-way ANOVA, followed by Tukey’s multiple comparison test.

Pro-inflammatory chemokines were determined in hCMEC/D3 and hOEC cell cultures infected with ZIKV MR766 and PRVABC59 at an m.o.i. of 1. At 24 h p.i. cell culture media were collected, clarified by centrifugation and used for the detection of CCL2, CCL3, CCL4, CCL5 and CXCL8 using ELISA. All chemokines were significantly upregulated in the samples from infected cultures compared to mock-infected cultures (Fig. 8). In particular, all these chemokines were produced at slightly higher levels in MR766-infected cells compared to cells infected with PRVABC59; however, these differences did not reach statistical significance.

## Discussion

The recent outbreak of ZIKV associated with neurological complications and microcephaly in newborns has stimulated a significant amount of research to investigate cell tropism, to develop suitable *in vitro* and *in vivo* models and to understand the mechanisms of ZIKV-induced pathology. Both Asian and African strains of ZIKV have been shown to replicate in a variety of human cell types, including epidermal, neural and placental cells, although there are differences in replication efficiencies, the type of cellular immune response induced and the induction of apoptosis and autophagy [26–32]. The African strain MR766 is very heavily adapted for mouse neurons and multiple studies confirm a higher infectivity and replication rate for MR766 using *in vitro* models compared to strains belonging to Asian genotype.

A direct *in vitro* comparison of the infectivity of neural stem cells and cellular responses induced by strains belonging to African and Asian genotypes suggested that the African strain had a higher infection rate and induced stronger anti-viral responses compared with the strain belonging to the Asian genotype [33]. Although neurological complications have been more evident with the recent outbreak of ZIKV in South America, caused by the Asian genotype, it is unclear if there are strain differences in neuropathology and it is possible that the disease associated with strains of African genotype are less frequently confirmed and characterized. In the present study, the permissiveness of human and mouse OEC and hCMEC/D3 cells to African MR766 and Asian ZIKV, the kinetics of viral growth, the localization of virus proteins and dsRNA in the neural cells, and ZIKV persistence as well as cytokine responses were investigated.

As expected, Vero cells supported high levels of viral replication. However, ZIKV also replicated very efficiently in hCMEC/D3 cells, which is in accordance with other studies [18, 20]. It is suggested that this efficient ZIKV replication in endothelial cells is due to the specific receptor AXL present in these cells [20] and that a potential mechanism for ZIKV invasion of the CNS via the BBB involves affecting brain capillary permeability [18].

ZIKV infection and replication has previously been shown for neuroglia within the CNS radial glia [34], myelinating oligodendrocytes [35] and astrocytes [36], but never for OECs, the neuroglia of the primary olfactory nervous system. OECs surround and support the axons of olfactory sensory neurons as they extend from the olfactory mucosa into the olfactory bulb in the brain, forming the olfactory nerve. OECs are also present in the outer layer of the olfactory bulb termed the nerve fibre layer, where they are thought to mediate the organization of primary olfactory axons to their correct bulbar targets [37–40]. The olfactory nerve is a route by which certain bacteria and viruses can reach the brain [14, 41–43]. Thus, OECs provide a potential route for access of pathogens and consequently have also been shown to be the main innate immune cells in the olfactory nerve [11]. In this study, we clearly showed the permissiveness of this cell type to support ZIKV infection. Compared to Vero or hCMEC/D3, viral titres were lower in hOEC and mOEC, suggesting reduced replication efficiency in these cells.

Despite the detection of reasonably high viral titres in the growth medium of neuroglial cells, the FACS analysis of infected cells suggested that only a small percentage of the cells contained detectable amounts of ZIKV NS3. Nevertheless, IFA using antibody against dsRNA, a marker for viral genome replication, confirmed the permissiveness of the cell lines for ZIKV RNA replication. The localization of NS3 in the cytoplasm is classical for flaviviruses. Colocalization of dsRNA and NS3 with ER membrane marker indicates that ZIKV replication complexes are, as expected, localized at ER membranes. Identifying other proteins interacting with NS3 and the role of these complexes in the coordination of replication steps and pathways could provide a target for impeding or interrupting viral replication.

The infection of Vero cells resulted in readily visible CPE from 3 to 5 days p.i.; this coincided with a decline in cell viability. Interestingly, no CPE was detected in neuroglial cells. Some studies have given insights into the possibility of persistent ZIKV infection. ZIKV was shown to persist in primary human foetal neural progenitors for at least 1 month with no activation of cytokine responses despite some cell death occurring [33]. Recently, a study on Sertoli cells showed that even after 6 weeks, cells were producing virions, suggesting that Sertoli cells are a major reservoir of virus [44]. *In vivo* studies in rhesus monkeys provide evidence of ZIKV persistence in the CNS and lymph nodes [45]. Mladinich *et al*. [18] reported the presence of ZIKV in primary human brain microvascular endothelial cells and hCMEC/D3 cultures at 9 days p.i. without CPE development and suggested that these cells could be susceptible to persistent infection. Here, we confirmed that ZIKV indeed develops persistent infection in hCMEC/D3 cells; even after 2 months of infection ZIKV RNA is present in these cells at high copy numbers and production of infectious virions was observed. In contrast, no particle production was observed for persistently infected mOEC or hOEC cells. There are two possible explanations for the absence of virus production in these cell lines. It is possible that these cells harbour defective but replicating viral genomes and lost the ability to produce plaques. It may also be that it was not possible to detect virus produced in these cells using standard assays (plaque assay) as the yield of infectious virus was below the detection level of the plaque assay. These results suggest that brain endothelial cells may act as a reservoir for ZIKV and a potential route for virus to reach to the brain. Additionally, persistent infection could be detected after 2 months in hOECs, albeit to a lesser extent and in a ZIKV strain/genotype-specific manner. The fact that the African strain, MR766, is heavily adapted for neurons compared to recent strains of the Asian genotype might explain the observed advantage of MR766 in these types of studies.

Mladnich *et al*. reported persistent ZIKV infection of primary human brain microvascular cells and suggested the involvement of the ISG15/IFN pathway in persistence, similar to that reported for hepatitis C virus [18]. How ZIKV is able to sustain stable infection without cytotoxicity remains to be resolved. Acutely infected cells produced high levels of pro-inflammatory chemokines, including CCL2, CCL3, CCL4, CCL5 and CXCL8, which could contribute to CNS inflammation during ZIKV infection *in vivo* by promoting the recruitment of various leukocytes into the CNS. CXCL8 (IL-8) and CCL3 (MIP-1α) could potentially recruit neutrophils, whilst CCL4 (MIP-1β) may recruit monocytes and NK cells. In particular, CCL2 (MCP-1), induced by flavivirus infection in various cells [46–48], could drive the recruitment of bone marrow-derived CCR2^hi^ inflammatory monocytes into the CNS in neuronal infection, as it does in other flavivirus models, to promote immunopathology [48, 49]. In the case of foetal neuronal infection, however, the extent to which this would recruit foetal monocytes or even maternal inflammatory monocytes is unclear. Later in infection sustained CCL5 production would likely drive maximal recruitment of the adaptive virus-specific T cell effectors that could clear virus. All these chemokines are chemoattractant for innate immune cells, consistent with their induction by flaviviruses early in infection, but each chemokine also has multiple functions in addition to chemoattraction [50, 51]. Given that many chemokines engage with multiple receptors, and all of the receptors for pro-inflammatory chemokines engage with multiple chemokines, it is presumably the combination of different receptors on individual cell types, activated by the differential concentration of each chemokine produced, that finally determines what cells migrate to the infected tissues and how each cell responds.

Overall, the growth kinetics analysis, replication efficiency studies, localization studies and cytokine expression studies suggest that neuroglial and brain endothelial cells are susceptible to ZIKV infection. Specifically, the fact that both olfactory ensheathing neuroglial cells and brain microvascular cells could support ZIKV replication may relate to the ability of the virus to enter the CNS via the olfactory nerve or via the BBB. The persistent infection detected without demonstrable CPE suggests that neuroglial cells and brain endothelial cells could provide a useful model for further investigation of intrinsic mechanism(s) responsible for reduced viral entry, RNA replication and/or virion formation. Use of these models can further promote our understanding of ZIKV-induced neuropathology.
